# The Immunogenic SigA Enterotoxin of *Shigella flexneri* 2a Binds to HEp-2 Cells and Induces Fodrin Redistribution in Intoxicated Epithelial Cells

**DOI:** 10.1371/journal.pone.0008223

**Published:** 2009-12-09

**Authors:** Keith Al-Hasani, Fernando Navarro-Garcia, Jazmin Huerta, Harry Sakellaris, Ben Adler

**Affiliations:** 1 Australian Research Council Centre of Excellence in Structural and Functional Microbial Genomics, Monash University, Clayton, Australia; 2 School of Biomedical, Biomolecular and Chemical Sciences, The University of Western Australia, Nedlands, Australia; 3 Department of Cell Biology, Centro de Investigación y de Estudios Avanzados del Instituto Politécnico Nacional, Mexico City, Mexico; Charité-Universitätsmedizin Berlin, Germany

## Abstract

**Background:**

We have previously shown that the enterotoxin SigA which resides on the *she* pathogenicity island (PAI) of *S. flexneri* 2a is an autonomously secreted serine protease capable of degrading casein. We have also demonstrated that SigA is cytopathic for HEp-2 cells and plays a role in the intestinal fluid accumulation associated with *S. flexneri* infections.

**Methods/Principal Findings:**

In this work we show that SigA binds specifically to HEp-2 cells and degrades recombinant human αII spectrin (α-fodrin) *in vitro*, suggesting that the cytotoxic and enterotoxic effects mediated by SigA are likely associated with the degradation of epithelial fodrin. Consistent with our data, this study also demonstrates that SigA cleaves intracellular fodrin *in situ*, causing its redistribution within cells. These results strongly implicate SigA in altering the cytoskeleton during the pathogenesis of shigellosis. On the basis of these findings, cleavage of fodrin is a novel mechanism of cellular intoxication for a *Shigella* toxin. Furthermore, information regarding immunogenicity to SigA in infected patients is lacking. We studied the immune response of SigA from day 28 post-challenge serum of one volunteer from *S. flexneri* 2a challenge studies. Our results demonstrate that SigA is immunogenic following infection with *S. flexneri* 2a.

**Conclusions:**

This work shows that SigA binds to epithelial HEp-2 cells as well as being able to induce fodrin degradation *in vitro* and *in situ*, further extending its documented role in the pathogenesis of *Shigella* infections.

## Introduction

Enteric infections are a major cause of morbidity and mortality worldwide. *Shigella* infections alone result in over a million deaths annually [1]. Infections with *Shigella* typically commence with watery diarrhea often progressing to a bloody, mucoid diarrhea characteristic of bacillary dysentery. Most of the virulence genes that have been studied are involved in the ability of *Shigella* spp. to invade the colonic epithelium and spread from cell to cell, leading eventually to inflammation, ulceration and bloody diarrhea [2], [3]. These genes are encoded on large, closely related, laterally acquired virulence plasmids found in all *Shigella* spp. and in related enteroinvasive *E. coli* (EIEC) [4].

At least five autotransporters have been identified in *S. flexneri*. The autotransporter proteins of Gram-negative bacteria form a family of proteins that share a common mechanism for autonomous secretion. All such proteins possess an N-terminal signal sequence for secretion across the inner membrane, a secreted mature protein referred to as the passenger or functional domain and a dedicated C-terminal domain which forms an amphipathic β-barrel pore that allows passage of the functional domain across the outer membrane. Exported proteins may remain tethered to the cell surface or be released from the cell by proteolytic cleavage [5]. Members of a subfamily known as SPATE (serine protease autotransporters of *Enterobacteriaceae*) possess a highly conserved serine protease motif [5], although not all autotransporters are proteases [6]. SepA (SPATE) is borne on the large virulence plasmid and is believed to play a role in tissue invasion [7]. VirG/IcsA plays a critical role in the recruitment and polymerisation of actin, thus allowing intra- and intercellular spread [3]. Pic (SPATE) is able to cleave mucin and human complement and a *pic* mutant is less inflammatory in ligated ileal loops [8]. Sap [9], a homologue of Ag43 in *E. coli* [8], is a phase-variable protein that mediates biofilm formation in *S. flexneri* (H. Sakellaris, personal communication).

The *sigA* gene, which is carried on the *she* PAI of *S. flexneri* 2a, encodes a 140 kDa protein which is autonomously secreted from the cell as a 103 kDa processed form. Similar processing occurs in *E. coli* clones harbouring *sigA* [10]. SigA is a temperature-regulated serine protease capable of degrading casein; it shows no proteolytic activity for IgA. As with the autotransporters Tsh and Pic, bacterial cells transiently expressing SigA on the surface agglutinate erythrocytes (K. Al-Hasani, unpublished data), indicating a cell-binding activity that may be related to its role in pathogenesis. We have shown that SigA is cytopathic for HEp-2 cells, inducing morphological changes similar to, but less pronounced than, those induced by Pet, a related autotransporter toxin in enteroaggregative *E. coli*. SigA causes a “rounding up” of HEp-2 cells, consistent with disruption of the cytoskeleton, causing a loss in the integrity of the cell monolayer [10]. In contrast, SepA and Pic from *S. flexneri* 2a induced no morphological changes on cell monolayers [11]. We have also demonstrated that SigA plays a role in the intestinal fluid accumulation associated with *S. flexneri* infections; a *sigA* mutant induced 30% less fluid accumulation in ligated rabbit ileal loops than its wild type parent [10]. The mechanism by which this occurs is unknown, but is likely to be related to the cytopathic effect of SigA on epithelial cells. The Pet toxin which shares 58% identity with SigA, degrades components of the cytoskeleton, fodrin (spectrin analog)/spectrin, in HEp-2 cells and erythrocytes respectively [12]. Based on the similarities in sequence and action of SigA to Pet, it is likely that SigA acts on the same cellular substrate. In agreement with this hypothesis, this work shows that SigA binds to epithelial HEp-2 cells as well as being able to induce fodrin degradation *in vitro* and *in situ*, further extending its documented role in virulence.

## Materials and Methods

### Ethics statement

Animal experimentation was approved by the Monash University School of Biomedical Sciences Animal Ethics Committee. All protocols involving human studies were approved by the University of Maryland Institutional Review Board (IRB) and Good Clinical Practices (GCP) were followed. Informed written consent was obtained from volunteers.

### Bacterial plasmids, and media

The bacterial plasmids used in this study, pPBA1100 (vector only), pSBA479 (pPBA1100 harbouring the complete *sigA* gene), and pSBA493 (*sigA* mutant) were described previously [10]. Clone 18531 contains bp 2531–4689 of human αII spectrin was kindly provided by Paul A. Stabatch [13], cloned into the *Eco*RI site of the inducible bacterial expression vector pGEX-3X (Pharmacia, Uppsala, Sweden). *Escherichia coli* DH5α and BL-21 were grown in either Luria Bertani (LB) or 2xYT medium at 37°C with aeration. When antibiotic selection was necessary, the growth medium was supplemented with ampicillin (100 µg/ml), kanamycin (50 µg/ml), tetracycline (10 µg/ml), streptomycin (25 µg/ml), or spectinomycin (25 µg/ml).

### Protein purification and analysis

Bacteria containing pPBA1100, pSBA479 or pSBA493 were grown in 3 ml 2xTY with kanamycin at 37°C for 16 h. Broth cultures were diluted 1∶100 in fresh medium and grown at 37°C to an absorbance at 600 nm of 0.5 (approximately 3.5 h), and then were induced for 1 to 3 h with isopropyl-β-D-thiogalactopyranoside (IPTG). The supernatants were obtained by centrifugation at 8,500 × *g* for 22 min, and then were filtered through 0.22-µm-pore-size membrane filters (Corning, Cambridge, Mass.) and concentrated 100-fold in an ultrafree centrifugal filter device with a 50-kDa cutoff (Millipore, Bedford, Mass.). Concentrated proteins were quantified by the Bradford method, aliquoted and stored at -20°C. Proteins were also analyzed by sodium dodecyl sulfate (SDS-PAGE).

GST-fodrin was prepared as described previously [13] with some modifications. Briefly, overnight bacterial culture of clone 18531 was diluted 1∶100 in fresh medium, grown for 1 h, and then induced for 1–3 h with IPTG before harvesting by centrifugation. Lysis was achieved by sonication, to which Glutathione Sepharose 4B beads (Pharmacia) were added to the soluble fraction for affinity absorption at 4°C for 1 h, centrifuged for 3 min and washed three times with 1.5 ml of phosphate-buffered saline pH 7.2 (PBS). The pellet was then resuspended in 250 µl 50 mM Tris-HCl, pH 9.6, containing 10 mM reduced glutathione.

### Tissue culture assay

For all experiments, concentrated proteins were diluted into Minimum Eagle's Essential Medium (MEM), without serum, to a final volume of 300 µl per well (for eight-well LabTek slides). The cells were treated with either SigA (56 µg/ml) or equal volumes of concentrated supernatants from *E. coli* containing ΔSigA (pSBA493) or the empty vector (pPBA1100) for 3 h in a humidified atmosphere of 5% CO_2_–95% air at 37° C. The medium was then aspirated and cells were washed twice with MEM and once with PBS.

### Immunostaining

Cells were fixed with 3% paraformaldehyde–PBS for 20 min, washed, permeabilized by addition of 0.1% Triton X-100–PBS for 10 min, washed, blocked with 1% BSA-PBS for 20 min. Immediately, the cells were incubated with anti-spectrin α II antibody (Santa Cruz Biotech, Santa Cruz, Calif.) at 1∶75 dilution for 1 h. The antigen-antibody reaction was developed by using the secondary antibody fluorescein-labeled donkey anti-goat IgG (Rockland, Philadelphia, Pennsylvania) at 1∶250 dilution for 1 h. Finally the cells were stained with 0.05 µg/ml of TRITC-phalloidin (Sigma Chemical) for 20 min. Slides were mounted on Vectashield Mounting Medium covered with glass coverslips and examined under a Leica TCS SP2 confocal microscope.

### Binding assay

The binding assay for SigA was performed with HEp-2 cells. HEp-2 cells were prepared by diluting to 19,000 cells/ml using a hemocytometer to estimate cell density. 100 µl of cells were added to each well of a 96 well tissue culture plate (Cluster^96^, Costar) and incubated overnight at 37°C in 5% CO_2_. Wells containing sub-confluent cells and wells not containing cells (used as a negative control) were washed three times for 5 min, and blocked for 30 min with TBS-Tween and 1% BSA. Wells were washed three times for 5 min and SigA protein was added in 50 µl or 100 µl volumes and incubated for 2 h. Excess protein was washed off (3×5 min) and 100 µl of 1 x sample buffer was added to solubilize the protein. The tissue culture tray was then wrapped in cling film and boiled for 5 min. All washes and incubations were carried out on a shaker at room temperature.

### Degradation assay

Ten microliters (1 µg) of purified GST-fodrin were mixed with equal volumes of 2x digestion buffer (0.3 mM CaCl2, 10 mM DTT) containing 2 µg of SigA. Reactions were carried out at 37°C at different time points and stopped by the addition of 2x SDS sample buffer. Samples were analyzed by SDS-PAGE. To inhibit the activity of SigA, SigA was preincubated for 30 min at 37°C with 2 mM phenylmethylsulfonyl fluoride (PMSF).

### Immunoblot analyses

Protein samples were analyzed by SDS-PAGE [14], and proteins were either stained with Coomassie blue or transferred onto Immobilon™ membranes (Millipore) as described by Chapman et al. [15]. For SigA HEp-2 binding assay experiments, the membranes were probed with primary rabbit anti-SigA polyclonal antiserum (diluted 1∶2000) [10] and then with a peroxidase-conjugated anti-rabbit IgG (diluted 1∶5000) as the secondary antibody (Chemicon International Inc., Temecula, CA). Bound antibody was detected by chemiluminescence reagents (Amersham Bioscience). The proteins from the degradation assays were probed with anti-GST rabbit polyclonal serum (diluted 1∶500), kindly supplied by Lev Katz, Monash University, to detect GST-fodrin. A peroxidase-conjugated anti-rabbit IgG antibody (diluted 1∶500) was used as the secondary antibody (Chemicon InternationalInc., Temecula, CA). 4-chloro-1-napthol was used as the chromogenic detection reagent.

A serum sample collected from a Center for Vaccine Development (CVD) volunteer who participated in *S. flexneria* 2a challenge studies was the generous gift of Myron Levine (University of Maryland, USA). Detection of SigA-specific antibodies in human serum was evaluated by immunoblot analysis. An immunblot of concentrated supernatant extracts from *S. flexneri* 2a YSH6000T (wild-type parent), SBA1356 (*ΔsepA-Δshe*) and SBA1359 (*ΔsigA-ΔsepA-Δshe*) was probed with the human serum sample (diluted 1∶600), and then with a peroxidase-conjugated anti-human Ig (diluted 1∶800) as the secondary antibody (Chemicon International Inc., Temecula, CA). These strains have been described previously [10]. The presence of SigA was detected using the same procedure as that used to detect GST-fodrin.

## Results and Discussion

**Figure 1:**
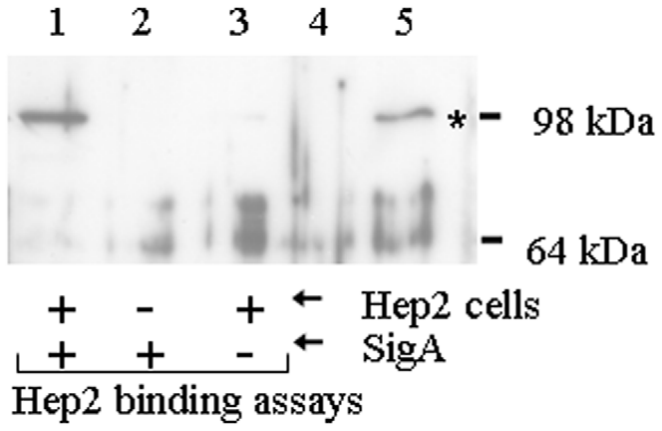
SigA binds specifically to HEp-2 cells. HEp-2 cells were incubated in the presence (lane 1) or absence (lane 3) of SigA and washed to remove unbound SigA. Bound SigA was recovered by treatment of HEp-2 cells with protein sample buffer and assayed by Western analysis with anti-SigA antiserum. A control for non-specific binding of SigA to the tissue culture plate was also included (lane 2). Culture supernatants of *E. coli* either not expressing (lane 4) or expressing (lane 5) SigA, indicate the position of SigA on the gel (asterisk). The positions of standard molecular mass markers (kDa) are shown on the right.

**Figure 2:**
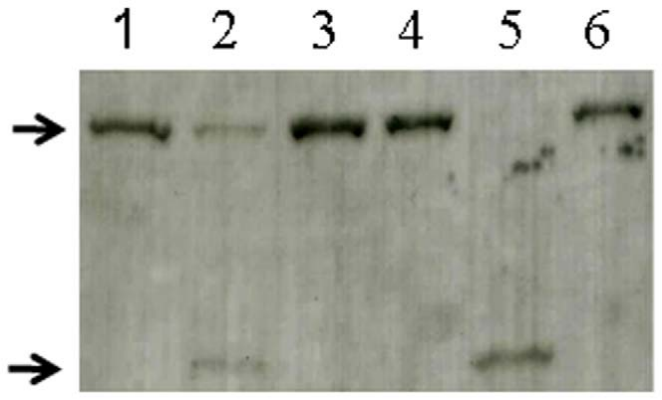
Degradation of human α-fodrin by SigA. Purified GST-fodrin was incubated with SigA (lanes 1, 2 and 3) or Pet positive control (lanes 4, 5 and 6) for zero (lanes 1 and 4) or 6 h (lanes 2 and 5). Lanes 3 and 6 shows SigA or Pet, respectively, pre-treated with PMSF and incubated with GST-fodrin for 6 h. The top arrow indicates the 109 kDa GST-fodrin fusion protein and the 74 kDa subproduct of degradation is indicated by the bottom arrow.

In this study, we sought to further characterize the biological role of SigA in the pathogenesis of shigellosis by investigating the nature of its interaction with the mammalian cell. SigA was incubated with HEp-2 cells, and bound SigA was detected by Western blotting using specific rabbit antiserum to SigA (Fig. 1, lane 1). SigA incubated in tissue culture wells in the absence of HEp-2 cells (Fig. 1, lane 2) was included as a control for non-specific binding to the tissue culture plate. No reaction band was seen in HEp-2 cells in the absence of SigA (Fig. 2, lane 3). PMSF-treated SigA bound equally well to HEp-2 cells, showing that proteolytic activity is not essential for cell binding (data not shown). This finding confirmed that the cell binding and the cytotoxic properties of this bifunctional molecule are distinct. The absence of correlation of the enzymatic activity of SigA with its binding activity has been noted for another related SPATE autotransporter, Tsh [16]. Those observations demonstrated that the passenger domain of Tsh is bifunctional, in that it exhibited both adhesive and proteolytic properties that are almost independent of each other.

To define the substrate specificity of SigA, we performed *in vitro* degradation experiments using recombinant α-fodrin, as the cloned fragment contains a protease hypersensitive region in the centre of α-fodrin [17]. GST-fodrin (109 kDa) was exposed to SigA protein preparation with or without PMSF. Western blot analysis showed that SigA degrades recombinant human αII-spectrin (α-fodrin) *in vitro* (Fig. 2), suggesting that the cytotoxic and enterotoxic effects mediated by SigA are likely associated with the degradation of epithelial cell fodrin. SigA protein preparations pre-incubated with PMSF did not demonstrate proteolytic activity for the recombinant GST-fodrin (Fig. 2, lane 6). The 72 kDa subproduct was detected by the anti-GST antibody, indicating that this fragment contains GST (27 kDa) and a α-fodrin fragment of 45 kDa. These data suggest that degradation of α-fodrin by SigA and Pet occurs at the same cleavage site within repeat 11 of α-fodrin. We were unsuccessful in our attempt to confirm the location of the cleavage by N-terminal amino acid sequencing of the putative 37 kDa sub-product from recombinant α-fodrin, due to our inability to visualize it by SDS-PAGE. We predict, however, that it occurs in the protease hypersensitivity region between M_1198_ and V_1199_ inside the calmodulin binding domain, based on previous work on Pet by Canizalez-Roman and Navarro-García [18]. Notably, complete degradation of recombinant α-fodrin was not observed even following overnight incubation with SigA (data not shown), whereas with Pet complete degradation of fodrin occurred within 2 h [18]. As was reported previously [10], when compared to SigA, purified Pet toxin, induced a more pronounced level of toxicity which may result from the differences in their abilities to degrade fodrin. The basis for this differential effect remains to be resolved but may be to due differences relating to their binding affinities to fodrin.

**Figure 3:**
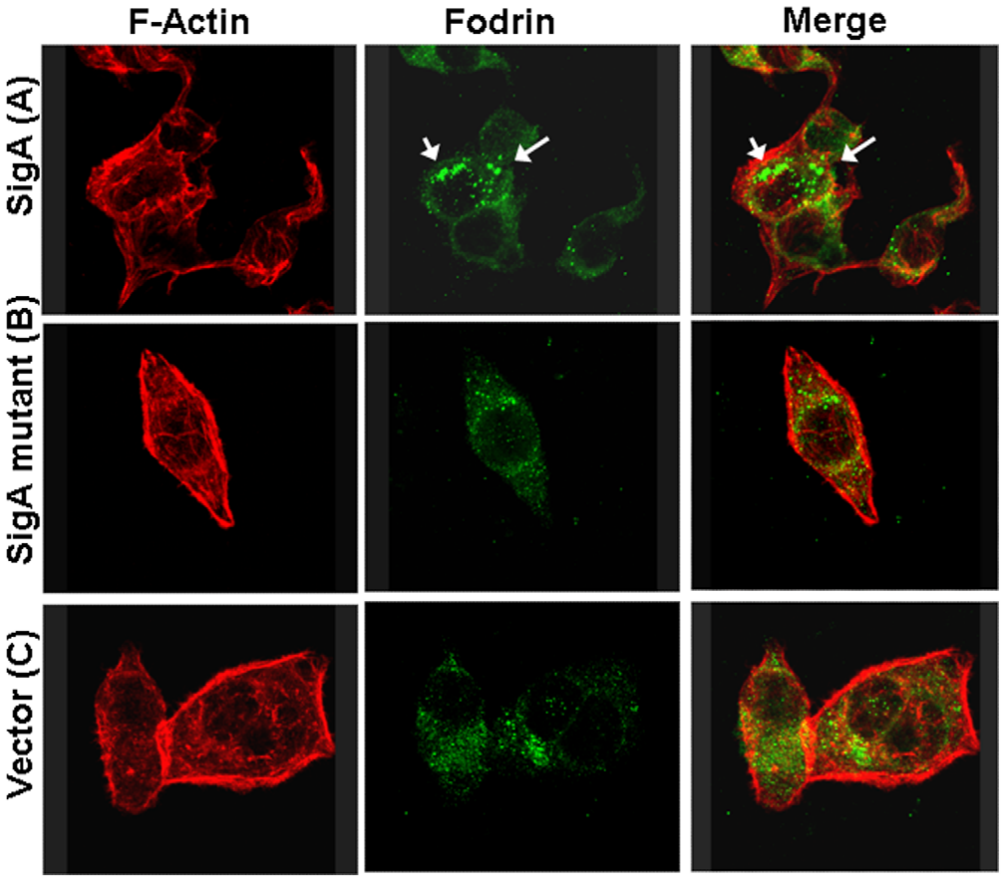
Effects of SigA on fodrin redistribution in epithelial cells. HEp-2 cells were treated for 3 h with supernatant from SigA clone (A), or ΔSigA (B) or vector only used as a control (C). Cells were then fixed and stained with rhodamine-phalloidin, and anti-α-fodrin antibodies, and a fluorescein-labeled secondary anti-goat antibody. Intracellular aggregates of fodrin are indicated by arrows.

It has been shown that Pet degrades fodrin, causing its redistribution in HEp-2 cells. Since SigA has affinity for recombinant α-fodrin, we investigated whether SigA was able to induce fodrin redistribution. To address this issue, HEp-2 cells were incubated with or without SigA or ΔSigA for 3 h, and the actin cytoskeleton and α-fodrin were stained with rhodamine-phalloidin and anti-α-fodrin antibodies and visualized by confocal microscopy. Staining of control cells (treated with supernatants from pPBA1100) with anti-fodrin antibodies revealed small green fluorescent spots along the epithelial cell (Fig. 3C). In contrast, a clear change in fodrin distribution was observed after 3 h of exposure with SigA (Fig. 3A). Fodrin was redistributed to form intracellular aggregates, which were located in membrane blebs, an indication that it may serve as an important substrate for SigA during the course of human infection. However, when cells were treated with the supernatant extract from the ΔSigA mutant, this characteristic redistribution was not observed and was comparable to the untreated cells (Fig. 3B). It is of interest that EspC, a SPATE autotransporter of EPEC, did not produce redistribution of fodrin in epithelial cells, as SigA and Pet do, suggesting differences among these closely related toxins [19]. These results do not exclude the possibility that additional cellular subtrates for SigA exist; thus the ability of SigA to proteolytically cleave other substrates warrants further investigation.

**Figure 4:**
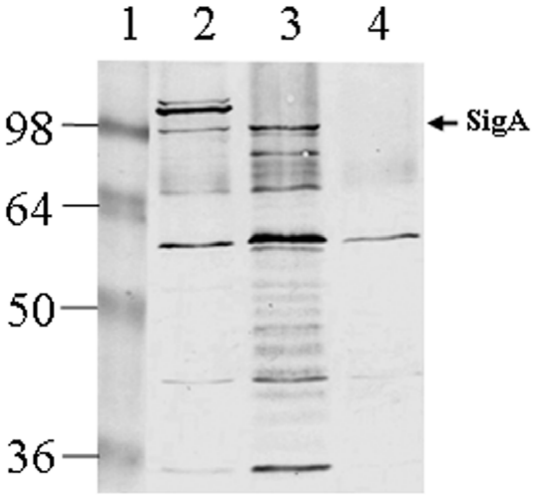
Antibody response against *S. flexneri* 2a SigA in a human volunteer. Concentrated culture supernatant extracts were separated by SDS-PAGE and immunoreacted with human post-challenge serum. Lanes: 1, molecular mass markers (kDa); 2, supernatant of *S. flexneri* 2a YSH6000T (parent strain); 3, supernatant of SBA1356 (*sepA* and *she* deficient double mutant); 4, supernatant of SBA1359 (*sigA-sepA-she* triple mutant). The arrowhead indicates the reactivity of serum from a volunteer against the 103-kDa secreted SigA antigen.

There is limited information concerning the nature and extent of the immune response to the SPATE family of autotransporters. Recently, there was a report on the detection of specific antibodies to two serine protease autotransporters, Pet and Pic, during the course of a natural EAEC infection in children [20]. To our knowledge, nothing is known about the *in vivo* expression of SigA during infection, and therefore, to gain a better understanding of the expression of SigA *in vivo*, we examined the antibody response against SigA with a volunteer infected with *S. flexneri* 2a. Concentrated culture supernatant extracts from the parental strain (*S. flexneri* 2a YSH6000T), SBA1356 (SepA and Pic deficient double mutant; previously described [10]), and SBA1359 (SigA, SepA and Pic triple mutant) were used in an immunoblot assay. The serum showed reactivity against three major bands with apparent molecular masses above 100 kDa, including native SigA (Fig. 4, Lane 2). In contrast, these three immunoreactive bands were not detected in the culture supernatant preparation of SBA1359 (previously described) which has all known *S. flexneri* 2a SPATE encoding genes (*sigA, sepA* and *she*) insertionally inactivated (Fig. 4, Lane 4). In the culture supernatant of SBA1356, a *sepA* and *she* defective strain, immunoreactivity with SigA was detected. However, the two immunoreactive bands which we suspect correspond to SepA and Pic (encoded by *pic(she)*) were absent. Normal human serum used as a negative control did not react against SigA (data not shown). To our knowledge, there are no published studies reporting on human antibody recognition of SepA. However, Pic from Enteroaggregative *E. coli* (EAEC) has been shown to be immunogenic [20]. Overall, this observation suggests the existence of antibodies against SigA, and possibly against all three *Shigella* SPATES. This serological investigation will provide the foundations for future work aimed at deciphering in more detail the host immune response against SigA, so as to eventually map the specific epitopes implicated in this induction as a prerequisite to the development of vaccines against shigellosis. Recently, Kotloff et al [21] inactivated the genes encoding the *Shigella* enterotoxins (ShET1 and 2) present on the *she* PAI and on the virulence plasmid respectively, and demonstrated in human clinical trials using this live, attenuated oral *Shigella* vaccine, their effectiveness in eliminating reactogenicity and reaching immune response levels that are likely to translate into clinical protection. This finding reinforces the need to comprehensively understand the biological relevance of the *she* PAI in establishing disease, in particular that of SigA, given the documented role of *sigA*. Taken together, this study clearly extends and further substantiates the role for SigA in the pathogenesis of *Shigella* infections.
